# Suppression of Hepatitis C Virus Genome Replication and Particle Production by a Novel Diacylglycerol Acyltransferases Inhibitor

**DOI:** 10.3390/molecules23082083

**Published:** 2018-08-20

**Authors:** Dahee Kim, Ja-Il Goo, Mi Il Kim, Sung-Jin Lee, Moonju Choi, Thoa Thi Than, Phuong Hong Nguyen, Marc P. Windisch, Kyeong Lee, Yongseok Choi, Choongho Lee

**Affiliations:** 1 College of Pharmacy, Dongguk University, Goyang 10326, Korea; gkdlfnwhel@nate.com (D.K.); aldlf998@dongguk.ac.kr (M.I.K.); flatronsky@gmail.com (S.-J.L.); ryche99@naver.com (M.C.); kaylee@dongguk.edu (K.L.); 2 School of Life Sciences and Biotechnology, Korea University, Seoul 02841, Korea; lighthil@gmail.com; 3 Hepatitis Research Laboratory, Department of Applied Molecular Virology, Institut Pasteur Korea, 696, Seongnam 13488, Korea; thoa.than@ip-korea.org (T.T.T.); phuong.nguyen@ip-korea.org (P.H.N.); marc.windisch@ip-korea.org (M.P.W.)

**Keywords:** hepatitis C virus (HCV), diacylglycerol acyltransferase (DGAT), lipid droplet (LD), DGAT inhibitor, HCV genome replication, HCV particle production

## Abstract

Diacylglycerol acyltransferases (DGATs) play a critical role in the biosynthesis of endogenous triglycerides (TGs) and formation of lipid droplets (LDs) in the liver. In particular, one member of DGATs, DGAT-1 was reported to be an essential host factor for the efficient production of hepatitis C virus (HCV) particles. By utilizing our previously characterized three different groups of twelve DGAT inhibitors, we found that one of the DGAT inhibitors, a 2-((4-adamantylphenoxy) methyl)-*N*-(furan-2-ylmethyl)-1*H*-benzo[d]imidazole-5-carboxam (**10j**) is a potent suppressor of both HCV genome replication and particle production. **10j** was able to induce inhibition of these two critical viral functions in a mutually separate manner. Abrogation of the viral genome replication by **10j** led to a significant reduction in the viral protein expression as well. Interestingly, we found that its antiviral effect did not depend on the reduction of TG biosynthesis by **10j**. This suggests that the inhibitory activity of **10j** against DGATs may not be directly related with its antiviral action.

## 1. Introduction

Chronic hepatitis C virus (HCV) infection is responsible for several inflammatory liver diseases such as liver cirrhosis and hepatocellular carcinoma [1]. HCV, a member of the *Flaviviridae* family, is a virus with a single-stranded RNA genome of positive polarity [2]. Upon entering hepatocytes via several liver-specific receptors, HCV expresses a single polyprotein composed of ≈3000 amino acids through a cap-independent translation of its RNA genome (≈9600 base pairs). The subsequent cleavage of this polyprotein by host and viral proteases results in production of three structural (core, E1 and E2) and six non-structural (NS) proteins (NS2, NS3, NS4A, NS4B, NS5A and NS5B) [3,4]. Since all of viral NS proteins play an essential role in the viral RNA genome replication, targeting their specific functions has been proven as an effective strategy to develop different kinds of anti-HCV therapeutics. 

Until recently, the standard of care (SOC) for chronic hepatitis C patients was based on combined treatment of PEGylated interferon (PEG-IFN)-α and ribavirin [5]. However, undesirable side effects including flu-like symptoms, anemia, depression and suicidal thoughts have been major concerns for this interferon-based combination therapy. Treatment with NS3 protease inhibitors (telaprevir and boceprevir)—the first direct-acting antivirals (DAAs) for HCV—were associated with less severe side-effects. With the second generation of DAAs like NS5A (daclatasvir and ledipasvir) and an NS5B inhibitor (sofosbuvir), the SOC for patients has shifted towards a triple combination regimen composed of one DAA plus PEGylated IFN-α and ribavirin [6]. Successful application of IFN-free combination treatment for 12 weeks using only ledipasvir (NS5A inhibitor) and sofosbuvir (NS5B polymerase inhibitor) has provided another treatment option to HCV patients depending on their infected viral genotypes [7]. However, in spite of their impressive high efficacy and good safety profiles, DAAs alone are not likely to play a central role in the next stage of HCV patient care because of their high financial burden, which will limit their access to the majority of patients chronically infected with HCV. In addition, many patients and social activists raised concerns for exorbitant high costs of DDAs. Therefore, a more affordable regimen for the treatment of HCV infection is still urgently desired.

Diacylglycerol acyltransferases (DGATs) are enzymes located at endoplasmic reticulum. They catalyze the final step in the biosynthesis of triglyceride (TG) through combination of acyl coenzyme A and diglyceride [8]. Two different kinds of DGATs including DGAT-1 and DGAT-2 have been shown to be directly involved in this biochemical lipid biosynthesis process. DGAT-1 is highly expressed in the small intestine, whereas DGAT-2 is primarily expressed in the liver [9]. Although they seem to perform a redundant task in TG metabolism in the hepatocyte, they were shown to play a critical role in overall secretion and deposition of TG. In addition, generation of sufficient amounts of TG is necessary for biogenesis of lipid droplet (LD) in the liver. Interestingly, LD was found to be a major site for HCV particle assembly and production [10,11]. Therefore, disruption of LD formation by various DGAT inhibitors has been envisaged as a plausible strategy to control HCV infection. However, in spite of its potent antiviral effect in vitro, the clinical trial of pradigastat—a commercially developed DGAT-1 inhibitor—was prematurely terminated due to lack of antiviral efficacy [12]. Its relatively high EC_50_ value in vitro (30 μM) and suboptimal pharmacokinetic profile might contribute to the failure of its clinical application [12]. Therefore, there is still a need to identify DGAT inhibitors with an improved antiviral efficacy and pharmacokinetic property.

In order to identify better DGAT inhibitors, we decided to utilize our DGAT inhibitor library composed of three different classes of twelve DGAT inhibitors based on their specificities against DGATs. We evaluated potential antiviral activities of three different classes of DGAT inhibitors [13,14,15]. As a result, we found that one of pan DGAT inhibitors, a 2-{[4-(adamant-1yl)phenoxy]methyl)-*N*-(furan-2-ylmethyl)-1*H*-benzo[d]imidazole-5-carboxamide (**10j**) possesses an antagonizing effect on both HCV genome replication and particle production and in a simultaneous manner.

## 2. Results

### 2.1. **10j** Suppresses Both HCV Genome Replication and Particle Production

**Figure 1:**
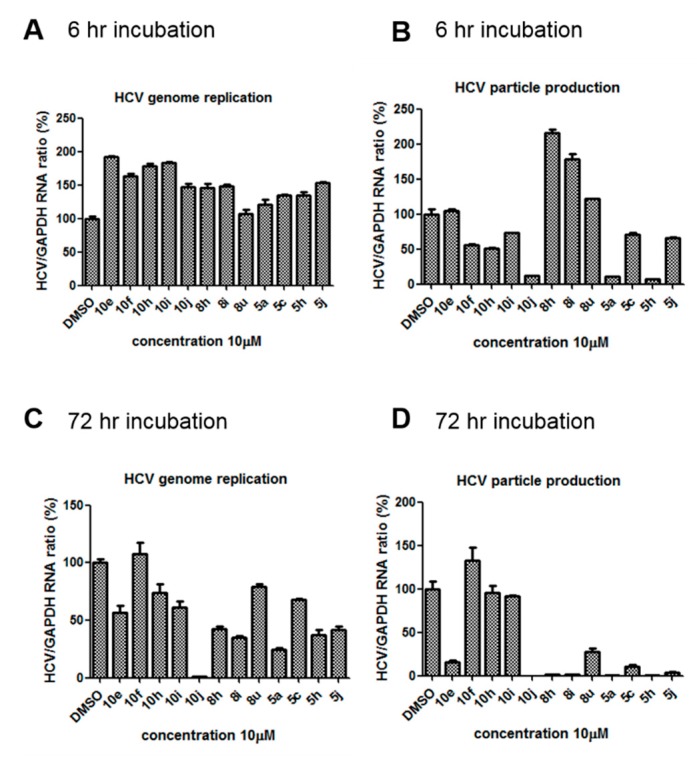
**10j** induces simultaneous blockage of both HCV replication and particle production. Huh7.5-J6/JFH1 cells were treated with twelve DGAT inhibitors at 10 μM for (**A**) 6 h and (**C**) 72 h. HCV and GAPDH RNA levels were measured by a real-time RT-PCR analysis. Supernatants collected from compound-treated Huh7.5-J6/JFH1 cells for (**B**) 6 h and (**D**) 72 h were incubated with naïve Huh7.5 cells to measure its infectivity by another round of a real-time RT-PCR analysis.

We previously synthesized and characterized twelve DGAT inhibitors with a different DGAT specificity [13,14,15]. As shown in Table 1, they include five pan DGATs inhibitors with a benzimidazole structure (**10e**, **10f**, **10h**, **10i**, and **10j**) [14], three DGAT-1-specific inhibitors with an indolyl hydrazides structure (**8h**, **8i**, and **8u**) [13] and four DGAT-2-specific inhibitors with an indolyl acrylamides structure (**5a**, **5c**, **5h**, and **5j**) [15]. Table 2 summarized their anti-DGAT activities and enzymatic specificities based on previously published results [13,14,15]. **A922500**, a commercially available DGAT-1-specific inhibitor was used as a positive control [10]. In spite of a wealth of knowledge on an essential role of DGAT-1 in the HCV particle assembly and production, less attention has been paid to potential functions of DGATs in other steps of the viral life cycle. Therefore, we decided to examine effects of these twelve DGAT inhibitors on HCV particle production as well as RNA genome replication in parallel. For this purpose, we transfected Huh7.5 cells with in vitro-transcribed genotype 2a HCV RNAs (Huh7.5-J6/JFH1) [16] and treated them with each DGAT inhibitor for 6 and 72 h at 10 μM. HCV genome replication as well as host GAPDH RNA levels was quantitated by a real-time RT-PCR analysis. For assessment of HCV particle production, we harvested and filtered the supernatant from Huh7.5-J6/JFH1 cells treated with DGAT inhibitors and incubated the supernatant with naïve huh7.5 cells for 72 h. Then, infectivity was measured by a real-time RT-PCR analysis. As shown in Figure 1A, all of DGAT inhibitors displayed no significant effect on HCV genome replication when incubated for 6 h. However, for the same period of time, three DGAT inhibitors including **10j**, **5a**, and **5h** were found to be able to block HCV particle production (Figure 1B). These data suggested that their antiviral activities were directed at late viral events such as assembly or packaging without affecting early viral events including the viral RNA genome replication. Interestingly when we performed the same experiment by using the 72-h incubation condition, we found that only **10j** was able to block both viral RNA genome replication and particle production in a simultaneous fashion (Figure 1C,D). Of note, all of three DGAT-1-specific inhibitors such as **8h**, **8i**, and **8u** and four DGAT-2-specific inhibitors such as **5a**, **5c**, **5h**, and **5j** were shown to suppress the viral particle production (Figure 1D).

**Figure 2:**
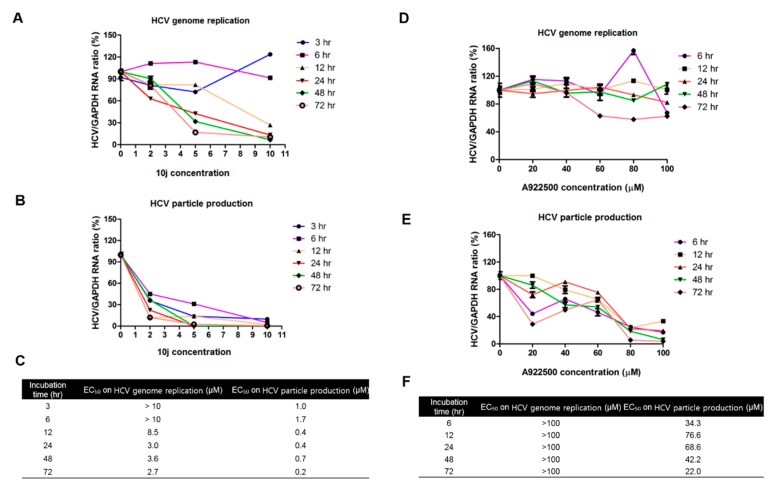
**10j** induces simultaneous blockage of both HCV replication and particle production in a mechanistically separate manner (**A**) Huh7.5-J6/JFH1 cells were treated with an increasing concentration of **10j** for a designated period of time (3, 6, 12, 24, 48, and 72 h). Then, their total RNAs were extracted and intracellular HCV RNA levels were measured by a real-time RT-PCR analysis. (**B**) In parallel, supernatants collected from **10j**-treated Huh7.5-J6/JFH1 cells were incubated with naïve Huh7.5 cells to measure its infectivity by another round of a real-time RT-PCR analysis. (**C**) EC_50_ values on HCV genome replication and particle productions were calculated based on data shown in (**A**,**B**). (**D**,**E**) Same experiments were performed as in (**A**,**B**) by using **A922500** except 3 h incubation. (**F**) EC_50_ values on HCV genome replication and particle productions were calculated based on data shown in (**D**,**E**).

For more detailed analysis of effects of **10j** on HCV particle production, Huh7.5-J6/JFH1 cells were treated with an increasing concentration of **10j** for designated periods of times (3, 6, 12, 24, 48, and 72 h). As shown in Figure 2A, treatment of **10j** for 3 and 6 h failed to show any significant effect on HCV genome replication up to 10 μM. However, when incubated for longer than 12 h, it started to induce dose-dependent suppression of HCV replication. Its EC_50_ values (effective concentration required to inhibit 50% of HCV replication) were 8.5, 3.0, 3.6, and 2.7 μM for 12, 24, 48, and 72 h, respectively (Figure 2C). In regard to the infectivity, **10j** was able to abolish the production of the infectious HCV particle regardless of the length of the incubation time (Figure 2B). Its EC_50_ values (effective concentration required to inhibit 50% of HCV particle production) were 1.0, 1.7, 0.4, 0.4, 0.7, and 0.2 μM for 3, 6, 12, 24, 48, and 72 h respectively (Figure 2C). When we conducted the same experiment by using a DGAT-1 inhibitor, **A922500**, we only observed loss of infectivity (EC_50_ = 22.0 μM for 72 h) with no significant effect on HCV replication (EC_50_ > 100 μM) (Figure 2D–F). These data suggest that **10j** is able to induce simultaneous blockage of both HCV RNA replication and particle production.

To order to study impacts of DGAT inhibitors on HCV genome replication independently of the particle production, we decided to examine effects of these twelve DGAT inhibitors on the levels of viral RNA genome and cell viability in parallel. For this purpose, we transfected Huh7.5 cells with in vitro-transcribed and luciferase-linked genotype 2a HCV RNAs [16] and passaged them for 3 weeks to confirm no significant production of HCV particles from these cells (data not shown). Then, we treated them with an increasing concentration of each DGAT inhibitor. Then, luciferase and MTT-based cell viability assays were performed to determine EC_50_ and CC_50_ (cytotoxic concentration required to kill 50% of treated cells) values, respectively. As shown in Table 3, three pan DGATs inhibitors including **10h**, **10i**, and **10j** were able to suppress HCV genome replication at a concentration below 10 μM. Especially, **10j** showed the most potent anti-HCV genome replication activity (EC_50_ 1.5 μM) with least cytotoxicity (CC_50_ 38.9 μM). It displayed the highest therapeutic index (TI), which was 24.6, among twelve DGAT inhibitors. Of note, its IC_50_ (inhibitory concentration required to inhibit 50% of enzymatic activities of DGATs) was reported to be 4.4 μM (Table 2), which was the lowest among five pan DGATs inhibitors [14]. On the other hand, TI values for three DGAT-1-specific inhibitors and four DGAT-2-specific inhibitors turned out to be lower than 3, indicating that most of their anti-HCV replication activities derived from their cytotoxicities. In addition, **A922500** also failed to show any anti-HCV genome replication activity (EC_50_ 50.8 μM and CC_50_ 71.9 μM) (Table 3). These data suggest that **10j** is the best HCV genome replication suppressor with an additional antiviral activity toward HCV particle production.

**Figure 3:**
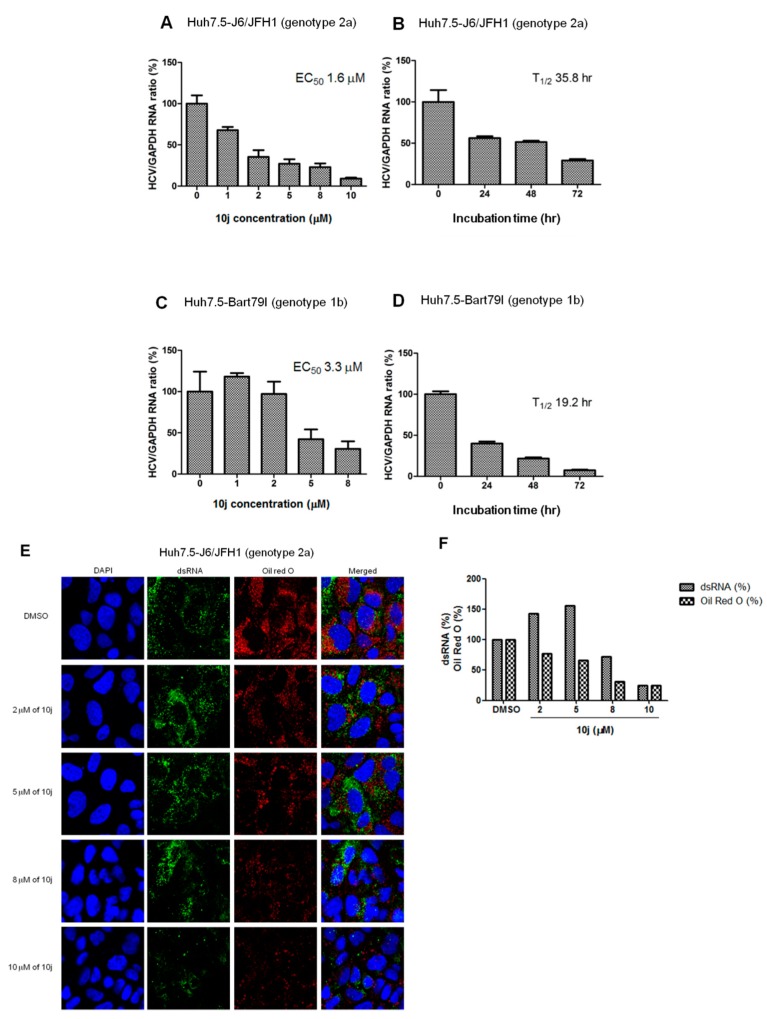
**10j** inhibits HCV replication in a reporter-free system. (**A**) Dose- as well as (**B**) time-dependent effects of **10j** on genotype 2a HCV genome replication was determined by measuring relative HCV as well as GAPDH RNA levels via a real-time RT-PCR analysis of Huh7.5-J6/JFH1 cells (**C**) Dose- as well as (**D**) time-dependent effects of **10j** on genotype 1b HCV genome replication was determined by measuring relative HCV as well as GAPDH RNA levels via a real-time RT-PCR analysis of Huh7.5-Bart79I cells. (**E**) Huh7.5-J6/JFH1 cells were treated with a series of increasing concentrations of **10j** for 72 h. Then, cells were stained with DAPI in blue for nucleus, an anti-J2 antibody in green for dsRNA and Oil-red-O in red for LD. (**F**) Quantitation of dsRNA and Oil red O signals shown in the Figure 3E.

**Figure 4:**
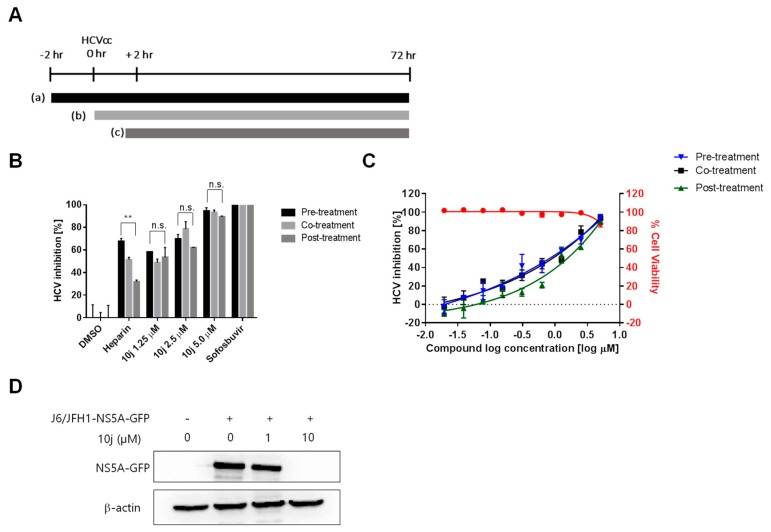
**10j** is required for late stage of HCV lifecycle. (**A**) Schematic representation of the time-of-addition experiment. Huh-7.5 cells were pre-treated for 2 h before HCVcc ((a) JFH1 virus expressing an NS5A-GFP) infection with **10j** at different concentrations or with 1% DMSO (solvent of compound), heparin (2 μg/mL), or sofosbuvir (10 μM) ((a) pre-treatment), or (b) co-treatment with HCVcc, or post treatment at 2 h after HCVcc infection ((c) post-treatment). (**B**) At 72 h post infection, HCVcc infection was measured by counting the number of GFP positive cells. *p*-Values are indicated by asterisks (** *p* < 0.01); Not significant (n.s.). (**C**) Determination of antiviral activity by dose response curve analysis. (**D**) Huh7.5 cells were infected with HCVcc and incubated with increasing concentrations of **10j** for 72 h. Expressions of NS5A-GFP proteins were quantitated by western blot analysis using a GFP antibody.

In order to rule out any artificial effects of the inserted luciferase on HCV replication, we decided to test the effect of **10j** on the reporter-free HCV genotype 2a infection clone (Huh7.5-J6/JFH1 (genotype 2a)) as well as sub-genomic replicon systems (Huh7.5-Bart79I (genotype 1b)). They were treated with a series of increasing concentrations of **10j** for 72 h. As shown in Figure 3A,C, both infectious clone (Huh7.5-J6/JFH1), as well as subgenomic replicon cells (Huh7.5-BART79I), produced similar ranges of EC_50_ values (1.6 μM and 3.3 μM, respectively) as compared with those determined by using the luciferase-linked replicon cells. They were also treated with 8 μM of **10j** for different periods of time to determine the length of time required for reducing the viral RNAs by half (T_1/2_). T_1/2_ of **10j** turned out to be 35.8 h for genotype 2a and 19.2 h for genotype 1b, respectively (Figure 3B,D). Since double-stranded (ds) RNAs were regarded as a genuine marker for endogenous viral RNA synthesis due to their roles as intermediates during viral RNA replication, we wished to test effect of **10j** on expression and localization of the viral dsRNAs. For this purpose, Huh7.5-J6/JFH1 cells were treated with a series of increasing concentrations of **10j** for 72 h. Then, dsRNAs were visualized by using a dsRNA-specific J2 antibody. As shown in Figure 3E, amount of dsRNAs was significantly reduced by treatment of **10j** in a dose-dependent manner. To study effects of **10j** on localization of LDs in parallel, LDs were also co-stained in an Oil-Red-O (ORO) dye. As shown in Figure 3E,F, expression level of total LDs also exhibited a dose-dependent reduction upon treatment of increasing concentrations of **10j**. In order to test the effects of **10j** on the early viral functions, such as the viral entry and uncoating of the viral genome, we performed the “time of addition” experiment by using the cell culture-derived infectious HCVcc particles (JFH1-NS5A-GFP) (Figure 4A). As shown in Figure 4B,C, infections of Huh7.5 cells with these HCVcc particles were all abrogated by pre-treatment (−2 h), co-treatment (0 h), as well as post-treatment of **10j** (+2 h) in a dose-dependent manner (Figure 4). These data suggest that **10j** does not seem to affect the early viral functions. Overall, these data suggest that **10j** is a potent anti-HCV replication as well as anti-TG synthesis inhibitor.

### 2.2. **10j** Reduces Expression Levels of HCV Proteins

**Figure 5:**
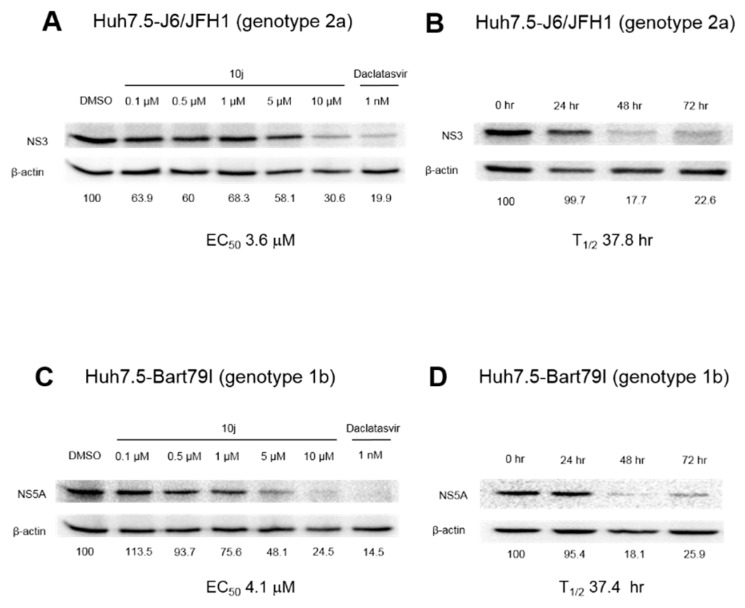
**10j** reduces expression levels of HCV proteins. (**A**) Dose-dependent western blot analysis of Huh7.5-J6/JFH1 cells treated with increasing concentrations of **10j** for 72 h by measuring protein expression levels of HCV NS3 relative to the *β*-actin. Daclatasvir, an NS5A inhibitor was used as a positive control. (**B**) Time-dependent western blot analysis of J6/JFH1 RNA-transfected Huh7.5 cells treated with 8 μM of **10j** for increasing periods of time analyzed by measuring protein expression levels of HCV NS3 relative to the *β*-actin. (**C**,**D**) Same experiments were performed as in (**A**,**B**) to study effects of **10j** on the expression levels of the NS5A protein of Huh7.5-Bart79I cells in a dose- as well as time-dependent manners.

Due to the tight coupling of viral genome replication to its protein expression, inhibition of viral RNA genome replication leads to a subsequent reduction of viral protein expression. In order to see if inhibition of HCV replication by **10j** translates into a loss of viral protein expression, we treated full-length genotype 2a (Huh7.5-J6/JFH1) as well as sub-genomic genotype 1b replicon (Huh7.5-Bart79I) cells with an increasing concentration of **10j**. As expected, we were able to see a dose-dependent decrease in expression levels of both HCV NS3 and NS5A proteins by western blot analyses (Figure 5A,C). Concentrations of **10j** required for reducing 50% of HCV protein expression turned out to be 3.6 μM for genotype 2a and 4.1 μM for genotype 1b, respectively. In addition, time required for reducing 50% of HCV protein expression in the presence of 10 μM of **10j** turned out to be 37.8 h for genotype 2a and 37.4 h for genotype 1b, respectively (Figure 5B,D). Daclatasvir, an NS5A inhibitor [17] was also able to inhibit expression of viral proteins as expected (Figure 5A,B). These data suggest **10j** is able to reduce the expression levels of viral proteins through blockage of the viral genome replication.

### 2.3. **10j** Suppresses the Biosynthesis of TG

**Figure 6:**
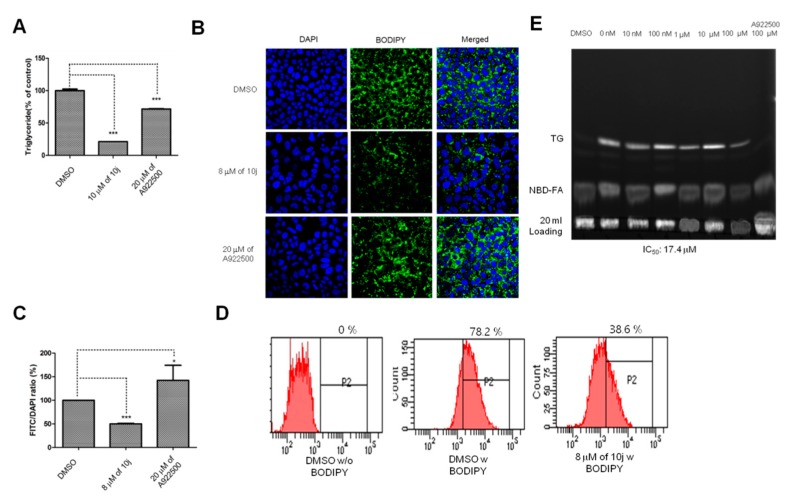
**10j** inhibits TG biosynthesis (**A**) Huh7.5 cells were treated with either DMSO, 10 μM of **10j**, or 20 μM of **A922500** for 72 h. Levels of endogenous TG were quantified with a TG quantification assay kit. (**B**) Huh7.5 cells were treated with either DMSO, 8 μM of **10j**, or 20 μM of **A9922500** for 72 h. Huh7.5 cells were stained with BODIPY and DAPI to visualize LDs and nucleus, respectively. (**C**) Levels of LDs shown in Figure 1B were quantified by using Image J software. (**D**) J6/JFH1 RNA-transfected Huh7.5 cells were treated with either DMSO or 8 μM of **10j** for 72 h. J6/JFH1 RNA-transfected Huh7.5 cells were stained with or without BODIPY and subjected to FACS analysis. Single asterisk (*) means *p*-value is between 0.1 and 0.5. Triple asterisk (***) means *p*-value is less than 0.01. (**E**) Dose-dependent inhibition of in vitro TG synthesis by **10j**. A DGAT-1 expression plasmid was transfected into 293T cells. Then, the DGAT-1 enzyme-containing membrane fractions were isolated from these transfected 293T cells. Membrane fractions were mixed with a fluorescent labeled NBD-palmitoyl CoA. Amount of newly synthesized TG was measured by a TLC plate in the presence of increasing concentrations of **10j**.

Once we confirmed the anti-HCV genome replication activity of **10j** in the previous experiments, we wonder if enzymatic suppression of DGATs by **10j** plays any role in the blockage of HCV genome replication. First, we tried to confirm the anti-TG biosynthesis activity of **10j**. For this purpose, we tested effects of **10j** on endogenous levels of TG in hepatocytes. As shown in Figure 6A, treatment of **10j** at 8 μM for 72 h was able to reduce the endogenous level of TG in Huh7.5 cells by 83% while only 25% of endogenous level of TG was suppressed by treatment of **A922500** at 20 μM. This result suggested that total TG level might be substantially decreased only by simultaneous blockage of both DGAT-1 and DGAT-2 enzymes. After confirming the **10j**’s ability to reduce the endogenous level of TG, we decided to test if **10j** is able to induce concurrent reduction in the formation of LDs, which are primarily composed of TG. As shown in Figure 6B, treatment of **10j** at 8 μM for 72 h was able to induce a 49% reduction in the level of LDs, which was visualized in green by an LD-specific BODIPY dye (Figure 6B,C). Interestingly, treatment of **A922500** at 20 μM for 72 h was able to result in a 45% increase in the level of LDs (Figure 6B,C). For more accurate quantification of intracellular levels of LDs, we performed FACS analyses in the absence or presence of 8 μM of **10j** for 72 h. As shown in Figure 6D, we observed a marked reduction in the relative number of LD-positive J6/JFH1 cells treated with **10j** (38.6%) when compared with those treated with DMSO (78.2%). We were also able to confirm the inhibition of in vitro synthesis of TG by DGAT-1 in the presence of **10j** with an IC_50_ value of 17.4 μM (Figure 6E). These data suggest that **10j** is able to suppress the biosynthesis of TG and subsequent formation of LDs.

### 2.4. Inhibition of HCV Genome Replication by **10j** Precedes Its Suppression of TG Synthesis

**Figure 7:**
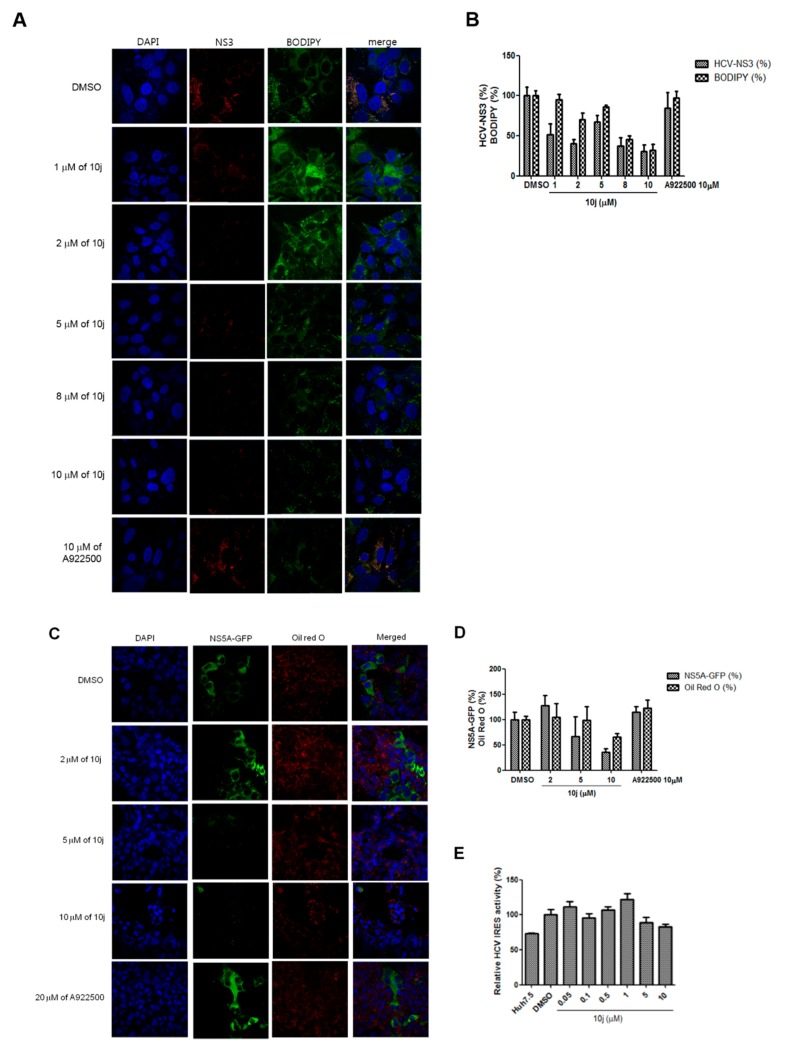
(**A**) Huh7.5-J6/JFH1 cells were treated with increasing concentrations of **10j** for 72 h. Cells were stained with an anti-NS3 antibody in red, DAPI in blue for nucleus and BODIPY for LD. (**B**) Relative percentages of NS3-and BODIPY-positive cells were quantified from more than three images from experiments shown in Figure 6A. (**C**) Huh7.5-JFH1-5A-GFP cells were treated with increasing concentrations of **10j** for 72 h. Cells were visualized in green for NS5A, DAPI in blue for nucleus and Oil Red O for LD. (**D**) Relative percentages of NS3-GFP- and LD-positive cells were quantified from more than three images from experiments shown in Figure 6C. (**E**) Effect of **10j** on HCV IRES-dependent translation. Huh7.5 cells were transfected with HCV IRES luciferase reporter plasmid followed by treatment of increasing concentrations of **10j**. HCV IRES-dependent translation efficiency was measured by a luciferase assay.

Based on previous results, we noticed that EC_50_ values required for inhibition of the HCV genome replication by **10j** were around 1–2 μM ranges (Figure 2 and Table 3). However, IC_50_ value required for inhibition of the enzymatic DGAT activities in vitro by **10j** was reported to be higher than 5 μM (Table 2 and Figure 6E). Based on these observations, we hypothesized that inhibition of DGATs by **10j** may not play a direct role in suppression of the viral genome replication. To test this hypothesis, we decided to study effects of **10j** on DGAT and the viral genome replication in a simultaneous manner. For this purpose, Huh7.5-J6/JFH1 cells were treated with a series of increasing concentrations of **10j** for 72 h. Then, the viral NS3 protein and LDs were visualized by using an anti-NS3 antibody and BODIPY, respectively. As shown in Figure 7A, the viral NS3 protein started to disappear at the concentration of 1 μM of **10j**, while the LD levels stayed unaltered by up to 2 μM of **10j**. Significant reduction of LD was only seen after treatment of **10j** higher than 5 μM. In order to confirm this observation, we decided to utilize another HCV-positive cell—JFH1-NS5A-GFP cells—which were infected with infectious HCV genotype 2a particles with a visually tractable NS5A due to the presence of in-frame-fused GFP protein with NS5A. TGs of these cells were stained with ORO for visualization in parallel. As shown in Figure 7C, **10j** treatment was able to reduce the expression of the NS5A-GFP in a dose-dependent manner starting at 5 μM. In accordance with the previous result, reduction of the NS5A-GFP protein preceded decline of TG biosynthesis (Figure 7D). Of note, **A922500** failed to induce any significant changes in the abundance of either GFP-tagged NS5A proteins or ORO-stained LDs (Figure 7C,D). In addition, HCV IRES (internal ribosomal entry site)-dependent translation also remained unaltered in spite of treatment of **10j** up to 10 μM (Figure 7E). These data suggest that antiviral activity of **10j** is not directly related with its anti-TG biosynthesis activity.

### 2.5. Effects of **10j** on Levels of a Liver-Specific Marker Such as HNF4α and miR122

**Figure 8:**
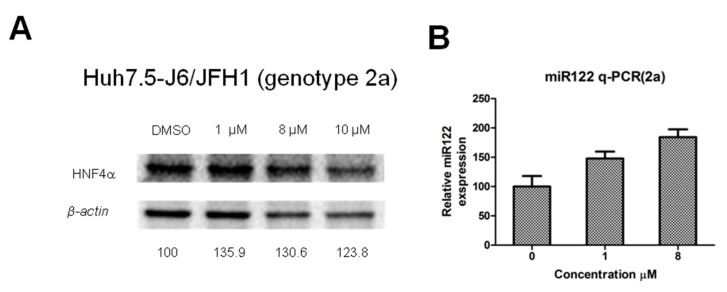
**10j** does not alter levels of liver-specific markers such as HNF4α and miR122. Huh7.5-J6/JFH1 cells were treated with a series of increasing concentrations of **10j** for 72 h. (**A**) HNF4α protein was relatively quantified to β-actin by western blot analysis. (**B**) miR122 was relatively quantified by RT-PCR analysis in the presence of increasing concentrations of **10j** in Huh7.5-J6/JFH1 cells.

Previous data strongly suggested that **10j** possesses the ability to abrogate HCV replication as well as viral particle production independent of disruption of DGATs. Since DGATs play an important role in maintaining liver-likeness in hepatocytes, the antiviral effect of **10j** might be due to mere loss of this liver-specific character. In this regard, we paid attention to hepatocyte nuclear factor 4a (HNF4α), which has been shown to be important for development and maintenance of hepatocyte [18]. In addition, HNF4α was also reported to be reduced in DGAT1-silenced cell lines. Therefore, we wished to test effects of **10j** on HNF4α. As shown in Figure 8A, expression levels of HNF4α remained unaltered despite treatment of **10j** up to 10 μM in Huh7.5-J6/JFH1 cells. In addition, expression levels of another liver-specific marker, miR122 did not decrease upon treatment of **10j** up to 8 μM in Huh7.5-J6/JFH1 cells (Figure 8B). These data suggest that anti-HCV replication activity of **10j** is not due to any negative effects on liver-specific characteristics of HCV host cells by **10j**.

## 3. Discussion

In this study, we found that **10j**, one of pan DGATs inhibitors with a benzimidazole moiety, is able to suppress HCV particle production as well as genome replication (Figure 2 and Table 3). Its anti-HCV replication activity was further verified by a significant decrease in the levels of HCV RNAs in both genotype 2a and 1b HCV RNAs-transfected cells (Figure 3). The negative effect of **10j** on HCV replication was also demonstrated by reduced subcellular localization of viral RNA replication intermediates, dsRNAs in **10j**-treated cells (Figure 3E). Blockage of HCV replication by **10j** was further translated into a decrease in expression levels of viral proteins including NS3 and NS5A (Figure 5). All these data strongly suggest that **10j** could serve as a promising antiviral candidate for HCV infection through negative modulation of two critical steps of the viral life cycle.

Initially, we noticed the presence of tight correlation of DGAT inhibitors’ potencies (IC_50_ values) and their anti-HCV replication activities (EC_50_ values) (Table 2 and Table 3). Therefore, we interpreted this finding as potential evidence for the strong dependency of HCV replication on the intact production of cellular TG by DGATs. However, 2.75-fold difference between IC_50_ (4.4 μM) and EC_50_ (1.6 μM) values of **10j** suggested no need for a complete shutdown of TG biosynthesis to initiate its suppressive activity against HCV replication. According to the previous study, knockdown of both DGAT-1 and DGAT-2 enzymes were necessary to significantly reduce neutral lipid content and LD numbers [11]. In regard to a potential role of LD in HCV replication, HCV NS4B protein was recently shown to target LDs through hydrophobic residues in the amphipathic helices to support HCV genome replication [19]. Therefore, provision of sufficient amounts of TG through DGATs-assisted biosynthesis might be necessary for construction of intact LDs in hepatocytes, which might be hijacked by HCV not only for its genome replication but also for infectious particle assembly. In this regard, pan DGATs inhibitory nature of **10j** might be critical to induce simultaneous disruption of both HCV genome replication and particle production. 

A possible explanation for the early reduction of HCV genome replication before reduction of LDs by **10j** could be deduced from following observations. A strong dependency of HCV on enzymatic activity of DGAT-1, not DGAT-2, for efficient production of HCV particles, was shown previously [11]. In their study, treatment of DGAT-1 inhibitor was able to suppress secretion of infectious HCV virions from infected cells. However, antagonism of HCV particle production by DGAT-1 inhibitor was not due to disruption of LD formation, since they failed to observe any change in overall neutral lipid content, LD size, or LD numbers in DGAT-1 inhibitor-treated cells [11]. In their subsequent study, they further showed that enzymatic activity of DGAT-1 is required for correct localization of NS5A to LD. In addition, they also found that only this correctly LD-localized NS5A is able to interact with the viral core protein to facilitate the efficient production of infectious HCV particles [10]. According to this study, DGAT-1 but not DGAT-2, was shown to play an essential role in HCV particle assembly. However, they failed to see any negative effects of **A922500** on HCV replication. In accordance with this, we also found little effect of **A922500** on either HCV replication or integrity of LDs (Figure 2D). We only observed its strong negative effect on the production of HCV particle (Figure 2E). However, in contrast to **A922500**, we were able to observe a marked reduction in the levels of HCV RNAs, proteins and LDs by treatment of our pan DGATs inhibitor, **10j** (Figure 3E). This suggests that simultaneous blockage of both DGATs enzymes might be necessary for complete down-regulation of LDs formation so that lowering amounts of LDs can induce an inhibitory effect on HCV replication. Redundant roles of DGAT-1 and DGAT-2 in TG synthesis further emphasize the necessity to block both DGAT-1 and DGAT-2 in order to shut down LD synthesis in a complete manner [20]. We hypothesize that overall integrity of LDs are essential for both HCV replication and particle assembly. Once defective LDs were generated by blockage of both DGAT-1 and DGAT-2 enzymes, they are no longer able to function as a proper platform for both HCV replication as well as viral particle assembly. However, according to our data, actual reduction of total amounts of LDs does not seem to be necessary for disruption of HCV genome replication. Therefore, **10j** seems to be able to induce a qualitative, not a quantitative, defect in the overall structures of LDs, which are no longer able to support HCV genome replication. It would be interesting to study the molecular mechanism for how the destruction of LDs by **10j** can lead to disruption of HCV replication complex in ER membrane in the future. 

One group of researcher reported impairment of HCV entry due to downregulation of claudin-1 in DGAT-1-deficient cells [21]. They found that the intracellular HCV RNA level was very low in the DGAT1-silenced cell lines, indicating that HCV RNA replication depends on the unknown function of DGAT-1. This study further supports a positive role of DGAT-1 in HCV replication. In this regard, their results were in line with our demonstration of inhibition of HCV replication by **10j**. However, this result seems to be inconsistent with the observation reported by Herker et al. [11]. They found no involvement of DGAT-1 in HCV replication. In regard to this discrepancy, we think transient inhibition of DGAT-1 for a few days may not be enough to induce any significant changes in the overall quantity and quality of LDs. Stable knockdown of DGAT-1, which was employed by Sung et al. might be necessary for full disruption of LDs [21]. In addition, due to the redundant roles of DGAT-1 and DGAT-2 in the biosynthesis of TG, simultaneous inhibition of both DGAT-1 and DGAT-2 may be needed for complete destruction of LDs in much earlier time. For this reason, our pan DGAT inhibitor, **10j** was able to succeed in impairing overall quantity and quality of LDs through dual blockage of both DGAT-1 and DGAT-2. This complete elimination of LD by **10j** eventually leads to inhibition of both HCV replication and particle production.

According to the previous study, the administration of the compound **10j** in mice resulted in decreased levels of total TG, total cholesterol and low-density lipoprotein cholesterol in the blood, accompanied by a significant increase in high-density lipoprotein-cholesterol [14]. This improvement in serum lipid profile by **10j** was comparable with the result of **A922500 [14]**. In this study, mice fed with high-fat diet and **10j** showed a significant decrease in all kinds of fat tissues in comparison with control mice [14]. This proven in vivo efficacy of **10j** in reducing total fat contents further demonstrates the utility of **10j** to develop as a new host-targeting antiviral candidate for the treatment of HCV.

Several compounds with a benzimidazole moiety were found to possess anti-HCV replication activity [22,23,24,25,26]. Most of them were shown to block exclusively HCV replication through inhibition of viral RNA-dependent RNA polymerase (RDRP), NS5B. Considering significant structural differences between **10j** and these benzimidazole-derived RDRP inhibitors, we do not believe any association of **10j**’s antiviral activity with RDRP. However, this possibility needs to be tested in the future. One compound with a benzimidazole stood out in terms of its antiviral mechanism of action due to its ability to disrupt HCV IRES-dependent translation [22]. When we tested the effect of **10j** on HCV IRES-dependent translation, we failed to observe any changes in HCV translation efficiency (Figure 7E). Therefore, we ruled out the possibility of **10j**’s inhibition of HCV replication through suppression of HCV translation. Of note, we found increased levels of miR122 by treatment of **10j** (Figure 8B). Dependence of HCV RNA replication on miR122 was well characterized [27]. Enriched level of miR122 was shown to assist the HCV RNA replication in the liver [27]. Therefore, we do not believe that increased miR122 by **10j** played any role in the inhibition of the HCV RNA genome replication by **10j**.

## 4. Materials and Methods

### 4.1. Cell Culture

Huh7.5 cells, which were subclones derived from Huh7 cells, are originated from hepatocarcinoma sample and were shown to support efficient HCV replication and production [28]. Huh7.5 cells were grown in monolayers as described previously [29,30]. Cell culture media contains DMEM (Sigma-Aldrich, St. Louis, MO, USA) supplemented with 1% l-glutamine (Hyclone), 1% penicillin, 1% streptomycin (Hyclone) and 10% FBS (JR Scientific, Woodland, CA, USA).

### 4.2. Plasmids

Rluc-J6/JFH1 (FL-J6/JFH-50C19Rluc2AUbi) is a HCV genotype 2a infectious clone described previously [16,31]. JFH-1-5A-GFP is an infectious genotype 2a HCV genome, which expresses the NS5A-GFP fusion protein [32]. Bart79I is a high-efficiency bicistronic subgenomic replicon of HCV obtained from the HCV genotype 1b Con1 sequence described previously [30]. FL-J6/JFH-50C19Rluc2AUbi and Bart79I were gifts from Dr. Charles Rice at Rockefeller University.

### 4.3. In-Vitro Transcription for Production of HCV RNA Genomes

In-vitro transcription for production of HCV RNA genomes was performed as previously described [33].

### 4.4. Generation of Stable HCV-Positive Cell Lines

The establishment of Huh7.5 cells, which stably maintain a Bart79I subgenomic replicon in the presence of G418 selection, has been described elsewhere [34]. The establishment of Huh7.5 cells, which stably maintain J6/JFH1, Rluc-J6/JFH1, or JFH1-NS5A-GFP HCV RNAs, was also previously described [35].

### 4.5. Cell Viability and Anti-HCV Replication Analysis Using a Luciferase Assay

Cell viability was measured with EZ-CYTOX (10% tetrazolium salt; Dogen, Gyeonggi-do, Korea) as described previously [36]. Anti-HCV replication analysis using a luciferase assay was conducted as previously described [35].

### 4.6. TG Quantification Assay

1.5 × 10^5^ of Huh7.5 cells were plated onto a 6-well plate (Costar 3610) and treated with either DMSO, 8 μM of **10j**, or 20 μM of **A922500** for 72 h. Cells were washed with cold PBS, suspended, and homogenized in 1 mL of 5% NP-40/ddH_2_O solution. Then, homogenized cells were slowly heated to 90 °C for 5 min or until the NP-40 becomes cloudy, then cooled down to room temperature. Previous step was repeated to solubilize all TG. Cells were centrifuged for 2 min at top speed using a microcentrifuge to remove any insoluble material. Samples were diluted 10-fold with ddH_2_O before proceeding with the assay. According to instructions included in the TG quantification assay kit (ab65336, Abcam, Cambridge, MA, USA), samples were added with 2 μL of lipase and incubated for 20 min at RT to convert TG to glycerol and fatty acid. Samples were added with 50 μL of reaction mix into each well and incubated at room temperature for 60 min while protected from light. Fluorescence was measured by using a fluorometric reader with Ex/Em = 535/590 nm.

The amount of TG from standard sample was calculated using the following equation:

$$\;\mathrm{Ts}=\frac{\mathrm{Corrected}\;\mathrm{absorbance}-\left(y-\mathrm{intercept}\right)}{\mathrm{slope}}\;$$

The amount of intracellular TG was calculated using the following equation:

$$\;\mathrm{Concentration}=\frac{\mathrm{Ts}}{\mathrm{Sv}}*D\;$$

where the Ts is the amount of TG from standard curve and Sv is the volume of sample added to sample wells and D is the sample dilution factor.

### 4.7. Visualization of Intracellular LD

Huh7.5 cells were incubated in a 24-well plate on top of coverslip in the presence of either DMSO, 8 μM of **10j**, or 20 μM of **A922500** for 72 h. Then, Huh7.5 cells were fixed by using 4% paraformaldehyde for 15 min and stained with 0.001% BODIPY 493/503 D3922 (Invitrogen, Carlsbad, CA, USA) for 10 min. The coverslips were mounted onto slides using Prolong Gold anti-fade reagent with DAPI (Invitrogen, Carlsbad, CA, USA) Fluorescence were examined and captured by Nikon confocal laser scanning microscopic system. LD content was quantified using the ImageJ program.

### 4.8. FACS Analysis

Huh7.5-J6/JFH1 cells were plated onto a 10 cc plate (Corning) and supplemented with either DMSO or 8 μM of **10j** for 72 h. Then, Huh7.5-J6/JFH1 cells were fixed by using 4% paraformaldehyde for 10 min and permeabilized using 0.1% saponin in PBS for 20 min. Huh7.5-J6/JFH1 cells were stained with 0.001% BODIPY 493/503 D3922 (Invitrogen) in saponin for 1 h at 4 °C. The FACSAriaIII (BD Biosciences, Franklin Lakes, NJ, USA) was used for the flow cytometric analysis as previously described [35].

### 4.9. Quantitative Real-Time RT-PCR Analysis

Quantitative real-time RT-PCR analysis to measure levels of HCV RNA and GAPDH was conducted as previously described [35]. The cDNA synthesis and reverse transcription of miR-122 were performed by using Taqman quantitative RT-PCR (qRT-PCR) analysis. Taqmanq RT-PCR components including TaqMan miR122 probe, endogenous control U6 probe, TaqMan^®^ MicroRNA reverse transcription Kit and TaqMan^®^ universal master mix II were purchased from Thermo Fisher Scientific (Waltham, MA, USA)

### 4.10. Immunofluorescence Analysis

Immunofluorescence analysis of Huh7.5-J6/JFH1 or Huh7.5-JFH1-5A-GFP cells in the presence of **10j** was conducted as previously described [35]. Fluorescence were examined and captured by Nikon confocal laser scanning microscopic system. LD content was quantified using the ImageJ program.

### 4.11. Western Blot Analysis

Western blot analysis of Huh7.5-J6/JFH1 or Bart79I cells treated with **10j** was conducted as previously described [35]. Relative amounts of HNF4α proteins were quantified by using an HNF4α mouse monoclonal antibody (M04), clone 4E2 (Abnova) with the dilution of 1:1000.

### 4.12. HCV Genome Replication and Particle Production Assay

Huh7.5-J6/JFH-1 cells were seeded into 6-well plates at a density of 3 × 10^5^ cells per well with DMEM containing 10% FBS. After 24 h, they are incubated with either DMSO, increasing concentrations of **10j** for 3, 6, 12, 24, 48, and 72 h, or increasing concentrations of **A922500** for 6, 12, 24, 48, and 72 h. Total RNAs were extracted from treated cells and virus-containing media were harvested at each time point. Naïve Huh7.5 cells were infected with these virus-containing media after concentration with centricons. Total RNAs were extracted from Huh7.5 cells infected with virus-containing media. qRT-PCR analysis was performed to measure effect of **10j** on HCV replication and virus particle production.

### 4.13. HCVcc Infectivity and the Time-of-Addition Assay

Cell culture adapted HCV JFH1 expressing an NS5A-GFP fusion protein was described previously [37]. The time-of-addition assay was conducted as previously reported [38]. Briefly, naïve Huh-7.5 cells were plated in 384-well plates and incubated with serially diluted **10j** at specific time points during virus infection: pre-infection treatment; co-infection treatment; post-infection treatment. Afterwards, infected cells were incubated at 37 °C for 3 days in the presence of inhibitors. At 72 h post-infection, HCV infection rates and cytotoxicity were determined by the number of GFP positive cells and Hoechst 33,342 positive cell nuclei, a marker for cell viability, respectively, using fully automated confocal microscopy (Opera, PerkinElmer, Hamburg, Germany). The acquired images were analyzed by in-house developed software. Percentages of HCV inhibition were calculated using 1% DMSO-treated cells and 10 μM of sofosbuvir treated cells and were set to 0% and 100% inhibition, respectively. Experiments were conducted at least two-times independently and in triplicates.

### 4.14. Total Membrane Isolation and DGAT-1 In Vitro Assay

pcDNA3.1-DGAT1 plasmid was transfected into 293T cells by using a lipofectamine 2000 transfection reagent (Invitrogen) as described by the manufacturer. At 24 h after transfection, transfected cells were harvested and cells were resuspended with 500 μL of 50 mM Tris-HCL/250 mM sucrose. Resuspended cells were disrupted by 15 passages through a 27-gauge needle and centrifugation at 600× *g* for 5 min. The supernatant was ultracentrifuged at 10,000× *g* for 30 min and the supernatant was discarded. The pellets were resuspended in a total of 500 μL of 50 mM ice-cold Tris-HCl/250 mM sucrose [39]. Then, a master mix containing 20 μL of 1 M-Tris-HCl, 4 μL of 1 M MgCl_2_, 10 μL of 4 mM DOG in acetone, 10 μL of 12 mg/mL BSA and 10 μL of 500 μM NBD-palmitoyl CoA in 20 mM Tris was prepared and incubated at 37 °C for 2 min. Also, 50 μg total membrane proteins were incubated with **10j** and **A922500** at 37 °C for 1 h. Extracted proteins were added into master mix and incubated at 37 °C for 10 min. The reaction was terminated by adding 4 mL CHCl_3_/methanol (2:1, *v*/*v*). Samples were mixed by vortexing and 800 μL of water was added and remixed. Samples were allowed to sit at room temperature for 1 h. Next, the TLC plate was developed in the solvent system with hexane/ethyl ether/acetic acid (80:20:1, *v*/*v*/*v*). Plate was allowed to air dry for 1 h before quantification of reaction products. The synthesized TG was determined by the ChemiDoc imaging system (Bio-Rad, Hércules, CA, USA) [39].

### 4.15. HCV IRES-Dependent Translation

A dual luciferase reporter plasmid [40], which expresses firefly and renila luciferases through cap and HCV IRES-dependent translation, was transfected into Huh7.5 cells in the presence of **10j**. Dual luciferase assay was conducted as previously described [40].

### 4.16. Statistical Analysis

Values in graphs represent the mean and standard deviations of representative experiments performed in triplicate or quadruplicate using Prism v5.0c software. Calculated *p*-values, which were less than 0.05 when compared with a control, were considered statistically significant. Single asterisk (*) means *p*-value is between 0.1 and 0.5. Triple asterisk (***) means *p*-value is less than 0.01. The resulting data were fit to the Hill equation using Prism v5.0c software to calculate EC_50_ and CC_50_ values.

## 5. Conclusions

In summary, we identified a pan DGAT inhibitor that simultaneously interferes with HCV genome replication and viral particle formation. Due to the ever-changing nature of RNA viruses, current antiviral drugs for HCV infection will develop resistance in the near future. In general, host-targeting antivirals are less prone to virus-induced resistance. In this regard, our newly identified pan DGAT inhibitor, **10j** will serve as a great lead compound to develop as another new class of host-targeting anti-HCV therapeutics. Especially, its dual targeting ability to disrupt both DGAT-1 and DGAT-2 could be further employed to maximize its anti-HCV activity through simultaneous blockage of both HCV replication and particle production.

## Figures and Tables

**Table 1:** Classification, chemical structures, code names of twelve DGAT inhibitors used in this study. Five benzimidazole derivatives (**10e**, **10f**, **10h**, **10i**, and **10j**) are pan DGATs inhibitors, three indolyl hydrazide derivatives (**8h**, **8i**, and **8u**) are DGAT-1-specific inhibitors and four indolyl acrylamide derivatives (**5a**, **5c**, **5h**, and **5j**) are DGAT-2-specific inhibitors.

Species	Code	Chemical Stucture
Benzimidazoles (pan DGATs inhibitors)	**10e**	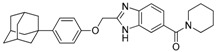
	**10f**	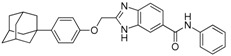
	**10h**	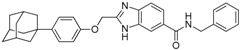
	**10i**	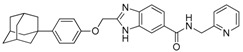
	**10j**	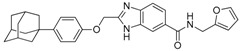
Indolyl hydrazides (DGAT-1 inhibitors)	**8h**	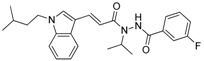
	**8i**	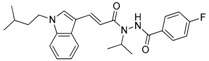
	**8u**	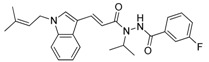
Indolyl acrylamides (DGAT-2 inhibitors)	**5a**	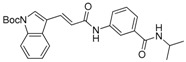
	**5c**	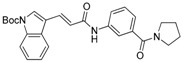
	**5h**	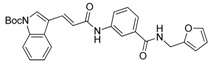
	**5j**	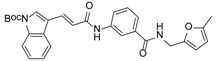
DGAT-1 inhibitor	**A922500**	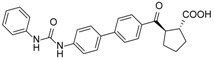

**Table 2:** Comparison of anti-TG synthesis activities of twelve DGAT inhibitors based on their enzymatic specificity. IC_50_ (RM) values were previously determined by using a rat liver microsome [14,15]. IC_50_ (DGAT-1) values were previously determined by using a recombinant DGAT-1 and [^14^C] triglyceride from [^14^C] oleoyl-CoA as an acyl-donor [13,15]. IC_50_ (DGAT-2) values were previously determined by using a recombinant DGAT-2 [13,15]. TG% (10 µM in HepG2) were previously determined relative [^14^C] triglyceride percentages when treated with 10 μM in HepG2 cells by using [^14^C] glycerol as a substrate [13,15]. N/D means not determined.

Species	Code	IC_50_ (RM) (μM)	IC_50_ (DGAT-1) (μM)	IC_50_ (DGAT-2) (μM)	TG % (10 μM in HepG2)
Benzimidazoles (pan DGATs inhibitors)	**10e**	27.5	N/D	N/D	N/D
	**10f**	>50	N/D	N/D	N/D
	**10h**	>50	N/D	N/D	N/D
	**10i**	20.0	N/D	N/D	N/D
	**10j**	4.4	9.0	17.3	45.0
Indolyl hydrazides (DGAT-1 inhibitors)	**8h**	N/D	20.4	>10	71.2
	**8i**	N/D	2.1	>10	94.8
	**8u**	N/D	1.5	>10	55.7
Indolyl acrylamides (DGAT-2 inhibitors)	**5a**	8.8	>100	6.9	N/D
	**5c**	13.2	89.1	6.8	N/D
	**5h**	2.5	>100	6.9	33.2
	**5j**	9.4	>100	7.4	N/D
DGAT-1 inhibitor	**A922500**	N/D	0.007	N/D	N/D

**Table 3:** Effects of twelve DGAT inhibitors on HCV genome replication. EC_50_ is an effective concentration required to inhibit 50% of HCV replication. CC_50_ is a cytotoxic concentration required to kill 50% of treated cells. Therapeutic index (TI) was calculated by dividing CC_50_ with EC_50_ values.

Species	Code	EC_50_ (μM)	CC_50_ (μM)	TI
Benzimidazoles (pan DGATs inhibitors)	**10e**	>10	>10	>1.0
	**10f**	>10	>10	>1.0
	**10h**	7.7	>10	>1.3
	**10i**	0.9	3.9	4.4
	**10j**	1.5	38.9	24.6
Indolyl hydrazides (DGAT-1 inhibitors)	**8h**	6.7	>10	>1.5
	**8i**	5.1	>10	>2.0
	**8u**	>10	>10	>1.0
Indolyl acrylamides (DGAT-2 inhibitors)	**5a**	1.9	3.7	1.9
	**5c**	>10	>10	>1.0
	**5h**	2.1	5.1	2.2
	**5j**	1.1	3.4	3.0
DGAT-1 inhibitor	**A922500**	50.8	71.9	1.4
